# Pleomorphic Adenoma of the Palate: Diagnostic and Surgical Strategy

**DOI:** 10.1155/2024/6400515

**Published:** 2024-08-06

**Authors:** Ciré Ndiaye, Mame Sanou Diouf, Houra Ahmed, Arame Thiam, Ngoné Diaba Diop, Aminata Mbaye, Mamadou Woury Barry, Mame Diarra Bousso Ba

**Affiliations:** ^1^ Department of Otolaryngology-Head and Neck Surgery Fann Teaching Hospital, Dakar, Senegal; ^2^ Department of Otolaryngology-Head and Neck Surgery Idrissa Pouye General Hospital, Dakar, Senegal; ^3^ Department of Pathology Fann Teaching Hospital, Dakar, Senegal

## Abstract

Pleomorphic adenoma is a benign tumor of the salivary glands. It develops preferentially in the parotid gland. The authors report a localization of a pleomorphic adenoma on the palate and discuss the value of CT scan in therapeutic strategy.

## 1. Introduction

Pleomorphic adenoma is a benign tumor of the salivary glands. It occurs predominantly in the parotid gland (80%), followed by the submandibular gland (10%) and the accessory salivary glands (10%) [1]. Among pleomorphic adenomas of the accessory salivary glands, localization to the palate accounts for 60%, followed by the upper lip 20% [2]. We report a case of pleomorphic adenoma of the hard palate and discuss diagnostic and therapeutic aspects.

## 2. Case Report

A 27-year-old female patient consulted the department of ENT for a swelling of the palate that had been evolving for 3 years, progressively increasing in volume without any troublesome symptoms.

Local examination revealed a hard, ovoid, painless mass of the palate with a firm consistency, approximately 3 cm long, with a healthy mucosa. There were no lymph nodes. The patient's general condition was good.

**Figure 1:**
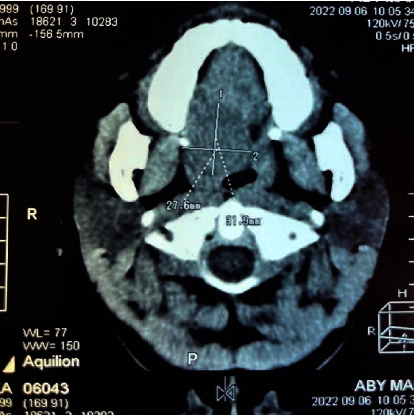
Axial-slice CT scan shows hypodense mass of the palate.

A CT scan revealed a dense, rounded mass located on the hard palate (Figure 1). There was no bone destruction. We did not perform fine needle aspiration. Because of the absence of clinical signs of malignancy, we opted for surgery.

**Figure 2:**
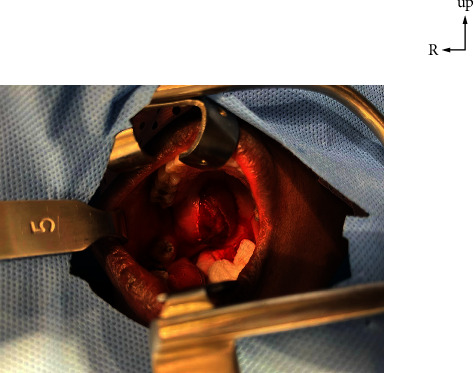
Mass of the palate, incision of the mucosa.

**Figure 3:**
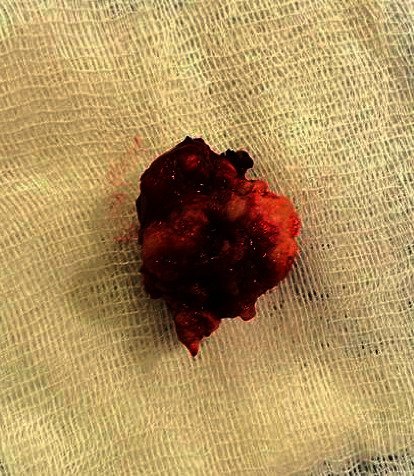
Specimen.

**Figure 4:**
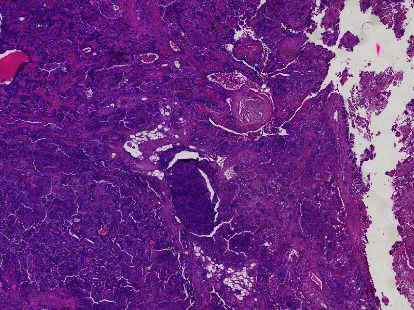
Pleomorphic adenoma of the palate: double contingent tumour proliferation, epithelial and conjunctival with cartilaginous metaplasia (hematoxylin and eosin, 40x).

The palate mass was removed under general anaesthesia (Figures 2 and 3). Postoperative follow-up was normal. Histology of the operative specimen showed a pleomorphic adenoma of the palate (Figure 4).

## 3. Discussion

Pleomorphic adenoma of the palate is a mixed benign tumor of the salivary glands of the palate mucosa. It represents 60% of pleomorphic adenomas of the accessory salivary glands. It occurs most frequently in women between the ages of 20 and 79 [3–6]. In the current study, the patient was younger, she was 27 years old.

The symptoms depend on the size of the tumor. When smaller than 6 cm, it is asymptomatic. At most, minor signs such as itching, chewing difficulties, or slight pain have been described [7].

Untreated, the size can exceed 9 to 10 cm and compromise respiratory, digestive, or phonatory functions, as reported by Andrea Bordoy-Soto et al. [2].

On physical examination, the pleomorphic adenoma typically presents as a smooth mass, firm in consistency, fixed in relation to the superficial and deep planes, covered by normal mucosa [3, 8–10].

Imaging like CT scan and MRI provides information on the exact location, size, surface and deep extension of the tumor [11]. CT scans are more accessible and can be used to study bone involvement. MRI better determines soft tissue extension.

Fine-needle aspiration cytology (FNAC) or trocar biopsy is necessary to orientate the diagnosis, with sensitivity levels of 75% and 100%, respectively [7].

Fine needle aspiration is often used to diagnose tumors of the main salivary glands. The oral cavity is easily accessible, so FNAC could be used to diagnose adenomas of the palate. Coupled with MRI, the two results would allow a histological orientation of pleomorphic adenoma. So Sunil et al. suggest that the main diagnostic methods are a combination of FNAC and imaging like CT scan and MRI [12].

Biopsy under local anaesthesia is more invasive than FNAC but allows a histological examination. Trocar biopsy can be reserved for cases where the results of cytology are indeterminate. This was the case of Antonios et al., who reported a case of pleomorphic adenoma of the palate which was not confirmed by the FNAC [11].

For the surgeon, having a cytological or histological orientation helps to adopt an appropriate surgical strategy.

Indeed, in terms of treatment, wide surgical excision, including the surrounding capsule, periosteum, and mucosa, is the treatment of choice for pleomorphic adenomas of the palate. Enucleation is not recommended because high recurrence rate are noted [12, 13].

In our patient, as in case of Zayd [14], we did not have a histological orientation, which is the reason why we preserved the mucosa adjacent to the mass, whereas resection of the mucosa is recommended to reduce the risk of recurrence. This attitude was adopted by some authors who had preoperative cytological or histological indications [13, 15].

Thus, to avoid recurrence, excision should be followed by curettage of the underlying bone using a sharp spoon or bur under copious irrigation with sterile normal saline [16]. However, bone curettage is not justified in the absence of bone erosion on CT scan, as in our patient's case. Patigaroo et al. described a case of maxillectomy for a palatal adenoma extending into the maxillary sinus [7]. CT scan is an indispensable tool for improving surgical strategy by studying bone involvement.

After resection of the adenoma, the defect on the palate can be repaired with a local flap or allowed to granulate [17].

At the end, we note that surgical attitudes are different from one surgeon to another.

Table 1 summarizes different surgical approaches. However, wide excision seems to be a consensus reported by all authors and postoperative follow-up is often simple.

## 4. Conclusion

Pleomorphic adenoma is a recurrent benign tumor, so long-term follow-up is essential. A preoperative cytology or histology and CT scan are essential for a better surgical strategy.

## Figures and Tables

**Table 1:** Summary of different surgical approaches.

Authors	Year	CT scan or MRI	Bone destruction	FNAC	Biopsy	Surgery approach	Postoperative follow up
Andrea Bordoy-Soto et al. [2]	2016	CT	Yes	No	Yes	Maxillectomy	Oral fistula
Gupta et al. [8]	2013	MRI	Yes	Yes	No	Wide excision with drilling bone	Simple
Tsekos et al. [11]		CT	Yes	Yes	No	Extend excision with flap reflection	Simple
Vasudev et al. [12]	2020	CT/MRI	No	Yes	No	Enucleation with curettage of the wall	Simple
		CT	No	Yes	No	Enucleation with curettage of the wall	
Zemmouri et al. [13]	2021		No	No	No	Enucleation	
Berrerhdoche et al. [14]	2022	CT	No	No	No	Enucleation	Simple
Hammami et al. [15]	2023	CT	No	Yes	Yes	Excision tumor and mucosa	Simple
Nnko et al. [18]	2023	CT	No	Yes	No	Enucleation	Simple
Panchal and Wanjari [19]	2023	No	No	No	No	Hemimaxillectomy with the reconstruction of the temporalis muscle flap	Simple
Fidêncio De Lima et al. [20]	2020	CT	No	No	Yes	Excision tumor and mucosa	Simple

## Data Availability

These patient's data are recorded in his or her medical file.
